# Nonlinear Kinetics and Isotherm Modeling of Rhodamine B Adsorption onto Al_2_O_3_ Decorated Laponite

**DOI:** 10.1021/acsomega.5c09190

**Published:** 2026-03-17

**Authors:** Lütfi Erden, İlyas Deveci, Hanife Erden

**Affiliations:** † Çanakkale Onsekiz Mart University, Can Vocational School, Department of Electricity and Energy, Çanakkale 17400, Türkiye; ‡ 531804 Konya Technical University, Vocational School of Technical Sciences, Chemistry and Chemical Processing Technologies, Konya 42250, Türkiye; § 52950 Çanakkale Onsekiz Mart University, Faculty of Engineering, Department of Chemical Engineering, Çanakkale 17100, Türkiye

## Abstract

The present study investigates the adsorption of Rhodamine B (RhB), a persistent cationic dye, onto Al_2_O_3_-decorated Laponite synthesized via a two-step modification process. In this method, cetyltrimethylammonium bromide (CTAB) was utilized to modify the Laponite surface, effectively preventing the gelation tendency of pristine Laponite and enhancing surface functionality. The characterization analyses yielded a BET surface area of 237.1 m^2^/g for the modified sample, in comparison to 319.0 m^2^/g for the unmodified Laponite. This was accompanied by an increase in mesopore volume (0.218 cm^3^/g vs 0.165 cm^3^/g). Experiments designed to optimize adsorption processes were conducted utilizing a Central Composite Design (CCD). The experimental outcomes demonstrated that under conditions deemed optimal (C_0_ ≈ 50 mg/L, dosage ≈ 30 mg, contact time ≈ 100 min), the maximum attainable removal efficiency for Rhodamine B (RhB) exceeded 92%. Kinetic analysis indicated that the Elovich model provided the most suitable fit (R^2^ = 0.994), thus confirming the heterogeneous and chemisorptive nature of the process. Among the isotherm models that were examined, the Toth model demonstrated the most optimal fit (q_max_ = 169.7 mg/g; R^2^ = 0.993), with the Sips and Redlich–Peterson models following closely behind, surpassing the performance of the Langmuir model (q_max_ = 156.9 mg/g; R^2^ = 0.991). Thermodynamic analysis revealed an endothermic adsorption process (ΔH = +11.16 kJ/mol), a positive entropy change (ΔS = +24.1 J/mol·K), and a range of ΔG values between +3.5 and +4.0 kJ/mol, suggesting a spontaneous tendency at higher temperatures. The findings of this study demonstrate that CTAB/Al_2_O_3_-modified Laponite is a highly effective adsorbent for the removal of RhB from wastewater.

## Graphical abstract

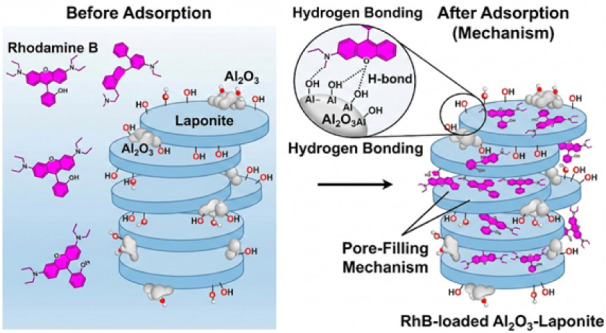

## 1 Introduction

Rhodamine B (RhB) is a water-soluble cationic dye that has found extensive application in the textile, paper and plastic industries.^1^ Even though its industrial use is increasing due to its high chemical stability, fluorescence properties and persistence, uncontrolled discharge of this compound into aquatic environments causes serious environmental and toxicological problems.^2^ Given the potential carcinogenic and mutagenic properties of RhB, its effective removal from water resources is imperative for environmental sustainability. Conventional treatment methods (e.g., coagulation, chemical oxidation, biological processes) frequently prove ineffective in the removal of persistent organic pollutants such as Rhodamine B. Consequently, adsorption-based advanced treatment methods are becoming increasingly important, particularly due to their selective, rapid and cost-effective nature.^3^

In this context, synthetic clays such as Laponite could be among the effective adsorbent candidates, thanks to their surface properties and ion exchange capacity.^4^ In recent years, considerable attention has been focused on the use of Laponite as an adsorbent for the removal of environmental pollutants (heavy metals, dyes, pharmaceuticals, etc.).^5^ This interest is attributable to the high cation exchange capacity (CEC) exhibited by Laponite, in conjunction with its substantial surface area and structural characteristics that are conducive to surface functionalization. Laponite has been demonstrated to enhance surface interactions and exert a favorable influence on the adsorption process, a property attributable to its capacity to generate stable colloidal suspensions in dispersion.^6,7^ However, its tendency to gel rapidly in aqueous media poses several challenges for its direct use in adsorption applications. This gelation has the effect of restricting mass transfer, especially in dynamic systems, and reducing process efficiency in steps such as adsorbent reuse or removal from the system. To overcome this problem, it is preferable to structurally transform Laponite into pillared clay and porous clay heterostructures (PCH)^5,8–10^ These structures are obtained by intercalating metal oxide precursors (e.g., Zr, Al, Fe) between the clay layers and then fixing them by appropriate heat treatment.^11,12^ This modification has been shown to prevent gelation by increasing the interlayer distance, while also significantly increasing the adsorption capacity by creating a permanent mesoporous structure. As evidenced by extant literature, the BET surface areas of Zr-columnar Laponite structures reach 400–500 m^2^/g.^8^ In addition, there is an increase in micropore volume and oxygen content, and these structures exhibit high adsorption capacity against organic pollutants such as Rhodamine B. Furthermore, in certain studies, the preadsorption of Laponite with molecules containing amine groups, such as ethylenediamine, has been observed to result in a reduction of interlayer agglomeration. This, in turn, has led to more homogeneous and open structures during the pillaring process. The utilization of such modified structures has yielded favorable outcomes in both the domains of physical adsorption and photocatalytic and redox-based environmental applications. For instance, in the removal of organic dyes (e.g., Rhodamine B, methylene blue), the performance of PCHs is enhanced not only by surface area but also by surface functionality and porosity. Surface functionalization with surface active agents or organosilane compounds has been demonstrated to provide selectivity and efficiency by strengthening the electrostatic and π-π interactions with the dyestuff.

In this study, a two-step modification strategy was applied to overcome the structural disadvantages of Laponite, such as its tendency to gel in aqueous media and limited interlayer accessibility. In the initial phase of the experiment, the interlayers of Laponite were modified with CTAB (cetyltrimethylammonium bromide), a cationic surfactant. CTAB neutralizes the surface charge on clay, thereby increasing interlayer hydrophobic interactions and creating an organophilic structure. This organomodification enhances the exfoliation capacity of the clay and establishes a conducive preliminary framework for intercalation. In the second stage, aluminum oxide (Al_2_O_3_) precursors were intercalated into the CTAB-modified Laponite interlayers to form a pillared clay structure. This process enabled the development of a porous structure by creating permanent and thermally stable inorganic pillars between the layers. Consequently, both the surface area and mesopore volume exhibited a substantial increase, while the propensity for gelation was effectively neutralized. The structural and morphological properties of the adsorbent obtained were extensively characterized in terms of surface morphology by means of SEM (Scanning Electron Microscopy), elemental composition by EDS, crystal structure by XRD (X-ray diffraction) and porosity and specific surface area by BET surface area analysis. These characterizations were conducted with the objective of verifying the effects of the modifications on the Laponite structure and of revealing the relationship with adsorption performance. To evaluate the efficiency of the modified Laponite-based adsorbents that had been obtained in Rhodamine B removal, experimental optimization was carried out depending on three independent variables. Contact time (minutes), initial concentration (mg/L) and adsorbent amount (g/L) were determined as independent variables; % removal rate was selected as the dependent variable (response). Consequently, a Central Composite Design (CCD) with three factors and three levels was employed. Furthermore, the impact of pH and temperature on the efficiency of removal was examined.

## 2 Experimental Section

### 2.1 Materials and Adsorbent Preparation

Synthetic Laponite RD (CEC ≈ 0.75 mequiv/g) was used as the host clay. Then, 37.5 mL of 0.1 M cetyltrimethylammonium bromide (CTAB) solution (corresponding to 100% of the cation exchange capacity) was added dropwise to the suspension. This step was performed to neutralize the surface charge and increase the hydrophobic character of the interlayer space. The resulting organoclay was filtered and washed with distilled water until the bromide ions were removed and dried at 60 °C overnight. The alumina precursor was prepared using the method described in the literature by Wang et al.^23^ For this purpose, 4.08 g of aluminum isopropoxide, Al­[OCH­(CH_3_)_2_]_3_, was dissolved in 100 mL anhydrous ethanol, followed by the addition of 4 mL concentrated nitric acid and 2.5 mL acetylacetone. The mixture was stirred in an oil bath at 70–80 °C for 1 h for controlled hydrolysis and sol formation. The CTAB-modified Laponite was dispersed under vigorous stirring in distilled water to a suspension of 2% by mass. For the intercalation process, preprepared alumina precursors were added drop by drop into the CTAB-modified Laponite mixture. The mixture was kept under continuous stirring for 24 h, the solid phase was filtered, washed again and dried at 80 °C. The resulting solid material was calcinated at 550 °C to obtain Al_2_O_3_ modified Laponite adsorbent.

### 2.2 Characterization

The surface morphology and elemental composition of the adsorbents were determined using a Scanning Electron Microscope (SEM, JEOL JEM-2100) equipped with an Energy Dispersive X-ray Spectroscopy (EDS) detector. The crystal structures were analyzed using an X-ray Diffractometer (XRD, EUROPE 600 Benchtop XRD) with Cu Kα radiation (λ = 1.5406 Å). The interlayer spacing (d) was calculated using Bragg’s equation:

$$d=\frac{\lambda }{2\sin\theta }$$

The specific surface area, pore size distribution, and pore volume were determined via N_2_ adsorption–desorption isotherms at 77 K using a surface area analyzer (Micromeritics TriStar II PLUS). The specific surface area was calculated using the Brunauer–Emmett–Teller (BET) method. Surface functional groups were investigated using Fourier Transform Infrared Spectroscopy (FTIR).

### 2.3 Experimental Design

Response Surface Methodology (RSM) was used to investigate the effects of three selected independent variables (concentration, adsorbent dosage and contact time) on the removal efficiency of the model compound. The experimental design was created with a 3-factor Central Composite Design (CCD) approach and planned by Minitab 21 software. A total of 20 experimental conditions were determined, and experimental error was estimated by means of repetitions performed at the central point. The independent variables were selected as initial solution concentration (5.0–100.0 mg/L), adsorbent dosage (10–50 mg/L) and contact time (5–180 min). During the experiments, the initial pH value, temperature (25 ± 1 °C) and stirring speed (150 rpm) were kept constant.

### 2.4 Batch Adsorption Experiments

Adsorption experiments were conducted under different conditions to determine the effects of parameters such as pH and temperature on Rhodamine B adsorption, to perform isotherm and kinetic studies. pH values were adjusted between 3 and 11 by using NaOH (0.1 M) or HCl (0.1 M), and temperature studies were conducted between 25 and 45 °C. A certain amount of adsorbent was added to 50 mL of dye solution and mixed for the specified times. After the reaction, the suspensions were centrifuged and the remaining dye concentration in the solution was measured with a UV–Vis spectrophotometer at a wavelength of 554 nm.

### 2.5 Kinetic, Isotherm, and Thermodynamic Modeling

#### 2.5.1 Adsorption Kinetics

To evaluate the adsorption mechanism and rate-controlling steps, the experimental data were analyzed using the nonlinear forms of the Pseudo-First Order (PFO), Pseudo-Second Order (PSO), and Elovich kinetic models. The equations for these models are expressed as follows:

The Pseudo-First Order (PFO) model:

$$q_{t}=q_{e}\left(1-e^{-k_{1}t}\right)$$

where q_t_ and q_e_ (mg/g) are the amounts of RhB adsorbed at time t and equilibrium, respectively, and k_1_ (1/min) is the rate constant.

The Pseudo-Second Order (PSO) model:

$$q_{t}=\frac{k_{2}q_{e}^{2}t}{1+k_{2}q_{e}t}$$

where k_2_ (g/mg·min) is the second-order rate constant.

The Elovich model, which is often used for chemisorption on heterogeneous surfaces:

$$q_{t}=\frac{1}{\beta }\ln\left(\alpha \beta t+1\right)$$

where α (mg/g·min) represents the initial adsorption rate and β (g/mg) is related to the extent of surface coverage and activation energy.

#### 2.5.2 Adsorption Isotherms

The equilibrium relationship between the adsorbate concentration in the liquid phase and the solid phase was analyzed using two-parameter (Langmuir, Freundlich, Jovanovich) and three-parameter (Toth, Redlich–Peterson, Sips) isotherm models.

The Langmuir isotherm assumes monolayer adsorption on a homogeneous surface:

$$q_{e}=\frac{q_{max}K_{L}C_{e}}{1+K_{L}C_{e}}$$

where q_max_ (mg/g) is the maximum adsorption capacity and K_L_ (L/mg) is the affinity constant.

The Freundlich isotherm describes multilayer adsorption on heterogeneous surfaces:

$$q_{e}=K_{F}C_{e}^{1/n}$$

where K_F_ represents the adsorption capacity and 1/n is the heterogeneity factor.

The Jovanovich model assumes monolayer adsorption but accounts for mechanical contacts between the adsorbate and adsorbent:

$$q_{e}=q_{max}\left(1-e^{-K_{J}C_{e}}\right)$$

The Toth isotherm is a semiempirical equation capable of describing heterogeneous systems at both low and high concentrations:

$$q_{e}=\frac{q_{max}C_{e}}{{\left(K_{T}+C_{e}^{t}\right)}^{1/t}}$$

where t is the heterogeneity parameter; deviations from unity indicate increased heterogeneity.

The Redlich–Peterson isotherm combines features of the Langmuir and Freundlich models:

$$q_{e}=\frac{AC_{e}}{1+BC_{e}^{\beta }}$$

where A, B, and β (0 < β < 1) are model constants.

The Sips isotherm predicts Freundlich behavior at low concentrations and Langmuir monolayer saturation at high concentrations:

$$q_{e}=\frac{q_{max}K_{S}C_{e}^{n}}{1+K_{S}C_{e}^{n}}$$

where K_S_ and n are the Sips equilibrium constant and heterogeneity exponent, respectively.

#### 2.5.3 Error Analysis and Thermodynamics

In the evaluation of model compatibility, instead of relying solely on the coefficient of determination (R^2^), a multidimensional comparison was conducted using various error functions: Total squared error (SSE), Chi-square (χ^2^), Hybrid error (HYBRID), and Mean absolute relative error (MARE). Additionally, the nature of the adsorption process was evaluated by calculating the thermodynamic parameters—free energy change (ΔG°), enthalpy change (ΔH°), and entropy change (ΔS°) - from the data obtained at different temperatures (298, 308, and 318 K).

## 3 Results and Discussion

### 3.1 Characterization

The structural, morphological, and surface properties of the pristine and Al_2_O_3_-decorated Laponite samples were evaluated comparatively to determine the effects of the modification and heat treatment.

The surface morphology changes were first examined using SEM. As shown in Figure 1a, the raw Laponite exhibits a smooth, continuous, and sheet-like surface structure typical of layered clay minerals. In contrast, the SEM image of the Al_2_O_3_-decorated Laponite (Figure 1b) reveals a significantly altered topography characterized by an irregular, clustered, and denser morphology. This distinct roughness indicates that the Al_2_O_3_ particles have successfully adhered to and decorated the Laponite surface, modifying its textural properties.

**Figure 1:**
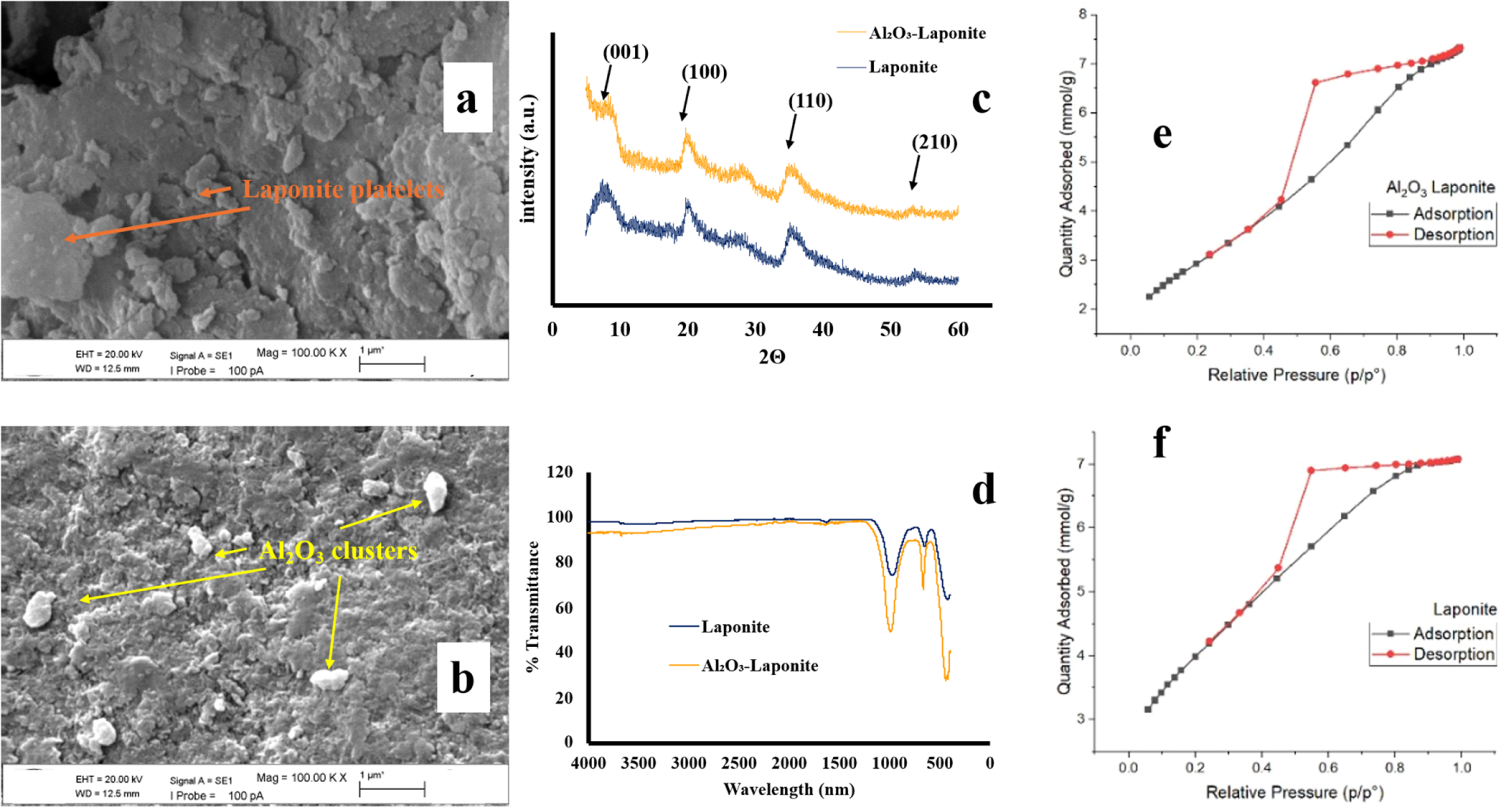
SEM image of raw Laponite (a), SEM image of Al_2_O_3_ decorated Laponite (b), XRD spectra of samples showing characteristic reflections corresponding to the (001), (100), (110), and (210) planes of the Laponite structure (c), FTIR spectra of samples (d) and N_2_ Ads/Des isotherms for Al_2_O_3_ decorated Laponite (e) and pristine Laponite (f).

To evaluate the impact of this surface modification on the crystal structure, XRD analyses were performed (Figure 1c). The strong reflection observed at approximately 2θ ≈ 7.45° in the raw Laponite sample corresponds to the typical (001) basal plane, indicating an interlayer distance of 11.86 Å. In the Al_2_O_3_-decorated Laponite, this peak shifted slightly to 7.69°, corresponding to a decreased d-spacing of 11.49 Å. This slight contraction indicates that the Al_2_O_3_ particles were immobilized primarily on the surface rather than intercalated deeply between layers, causing a limited rearrangement without disrupting the main crystal framework. Apart from the basal spacing, the diffraction peaks observed at 2θ ≈ 19.7°, 27.5°, 35.2°, and 53.0° correspond to the (100), (110), and (210) planes of the Laponite structure, respectively. The preservation of these peaks in the Al_2_O_3_-decorated sample indicates that the intralayer crystalline framework of the Laponite was not disrupted during the modification process.

FTIR analysis was conducted to identify the chemical changes and functional groups present (Figure 1d). In the spectrum of raw Laponite, a strong peak around 1000 cm^–1^ represents the asymmetric stretching vibration of Si–O–Si, while bands in the 500–600 cm^–1^ range correspond to Mg–O and Si–O bending modes. In the Al_2_O_3_-decorated sample, changes in the intensity and slight shifts in these bands were observed, particularly in the low-frequency region. These spectral variations confirm the immobilization of Al_2_O_3_ nanoparticles and the formation of new chemical interactions on the adsorbent surface.

The textural properties were further quantified using BET analysis (Table 1) and N_2_ adsorption–desorption isotherms (Figure 1e and f). Both samples exhibited Type IV isotherms with H3-type hysteresis loops, characteristic of mesoporous structures with slit-shaped pores. While the specific surface area decreased from 319.0 m^2^/g (pristine) to 237.1 m^2^/g (modified) due to partial pore blocking, the modification led to a beneficial rearrangement of the pore structure. The average pore width increased from 3.07 to 4.26 nm, and the cumulative mesopore volume increased by approximately 32%.

**Table 1:** Textural Properties of Laponite and Al_2_O_3_ Decorated Laponite

Textural Properties	Laponite	Al_2_O_3_ Laponite
Single point surface area at (m^2^/g)	310.8	229.1
BET surface area (m^2^/g)	319.0	237.1
t-plot micropore area (m^2^/g)	44.6	8.8
t-plot external surface area (m^2^/g)	274.4	228.3
Single point adsorption total pore volume of pores (cm^3^/g)	0.245	0.252
t-plot micropore volume (cm^3^/g)	0.018	0.002
BJH adsorption cumulative volume of pores (cm^3^/g)	0.166	0.218
Adsorption average pore width (4 V/A by BET) (nm)	3.07	4.26
EDS Analysis		
Element		
Oxygen	88.95	81.45
Silicon	6.22	10.44
Magnesium	4.17	6.64
Aluminum	-	1.47
Si/Mg	1.49	1.57
Si/Al		7.1

Structure–Property Relationship: This evolution in pore architecture is critical for the adsorption performance. The Rhodamine B molecule has large molecular dimensions (approximately 1.8 nm × 0.7 nm); therefore, its diffusion is often sterically hindered in microporous materials. The expansion of the average pore width to 4.26 nm effectively creates a mesoporous network that facilitates the intraparticle diffusion of the bulky RhB dye molecules into the internal active sites. Thus, even though the total surface area decreased, the accessible surface area for the large dye molecules was optimized.

Finally, EDS analysis confirmed the chemical integration of the modifier, revealing the presence of aluminum (1.47 wt %) in the decorated sample. The increase in the Si/Mg ratio (from 1.49 to 1.57) and the detection of Al further support the successful coating of the Laponite surface.

It should be noted that an apparent increase in the weight percentages of silicon and magnesium was observed following the modification (Table 1). This variation is primarily attributed to the removal of volatile organic components (such as residual CTAB and adsorbed water) during the calcination process at 550 °C, which results in a relative concentration of the inorganic framework elements in the final matrix. Additionally, the semiquantitative nature of EDS analysis, which can be influenced by surface roughness and interaction volume, may contribute to these observed shifts in elemental ratios.

### 3.2 Effect of Experimental Parameters on Adsorption Efficiency

In this study, the performance of an Alumina Decorated Laponite-based adsorbent system used for the removal of Rhodamine B (RhB) dye from aqueous solution was optimized using Central Composite Design (CCD). The effects of three key process parameters initial dye concentration, adsorbent amount, and contact time on % removal were evaluated using multivariate statistical analyses. The established regression model estimates the adsorption efficiency as follows:

$$\%\mathrm{Removal}=40.22-0.306C+2.159A+0.3042T-0.002997C^{2}-0.03229A^{2}-0.000472T^{2}+0.01722CA-0.000769CT-0.002685AT$$

Where C: initial concentration (mg/L), A: adsorbent amount (g), and T: contact time (min). In general, it was determined that maximum Rhodamine B removal efficiency can be achieved with a combination of high adsorbent amount, sufficient contact time, and low initial concentrations. The resulting quadratic regression model represented the experimental data with high accuracy, and the overall validity of the model was supported by strong statistical indicators (R^2^ = 98.69%, R^2^ (adj) = 97.50%, R^2^ (pred) = 91.62%). The standard error of the model, 3.45, demonstrates the reliability of its predictive power. Furthermore, the P = 0.086 obtained from the Lack-of-Fit test confirms that the model does not show a significant mismatch with the experimental data. ANOVA results of analysis are seen in Table S1.

According to the ANOVA results, the model’s linear, quadratic, and interaction terms were statistically significant (P < 0.05). Removal efficiency was positively influenced by an increase in adsorbent amount and contact time but was negatively affected by a higher initial dye concentration. The significant interactions between these parameters demonstrate their combined effect on the adsorption process.

#### 3.2.1 Interaction Effects and Optimization

The 3D contour plots (Figure 2a–c) provide critical insights into the interaction effects between the operating parameters, revealing the mechanistic behavior of the adsorption system.

**Figure 2:**
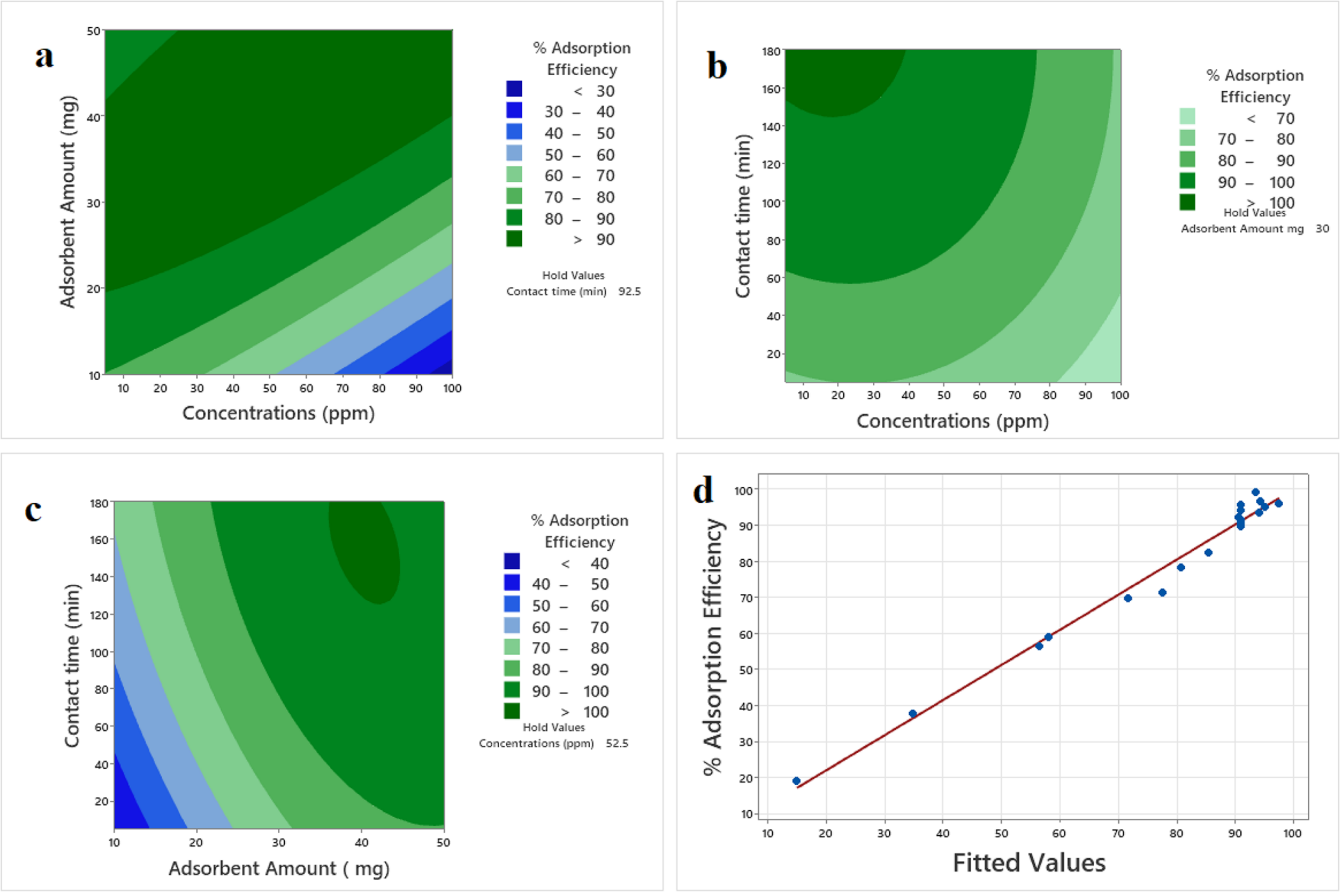
Contour plot of the effect of initial Rhodamine B concentration and adsorbent (Laponite) amount on % adsorption efficiency (fixed contact time: 92.5 min) (a); effect of initial concentration and contact time on % removal (fixed adsorbent amount: 30 g) (b); effect of the interaction between adsorbent amount and contact time on % adsorption efficiency (fixed concentration: 52.5 mg/L) (c); comparison of experimental and model-predicted % adsorption efficiencies (d).

Figure 2a illustrates the interaction between initial dye concentration and adsorbent dosage. The plot clearly shows that removal efficiency is maximized (>90%) in the region of high adsorbent dosage and low initial concentration. This behavior is governed by the ratio of available active sites to adsorbate molecules. At high dosages, the total surface area and the number of adsorption sites, specifically the Al–OH and Al–O groups introduced by the modification, are abundant relative to the number of RhB molecules. Conversely, as the initial concentration increases while dosage remains fixed, the finite number of active sites becomes rapidly saturated. The curvature of the contour lines indicates that the negative impact of increasing concentration is far more severe at low adsorbent dosages, where the system quickly reaches its capacity limit.

Figure 2b elucidates the relationship between contact time and initial concentration. A distinct interaction is observed: extending the contact time significantly enhances removal efficiency at lower concentrations, but this effect diminishes at higher concentrations. This can be attributed to the diffusion dynamics. At low concentrations, the driving force for mass transfer is lower, making the process more dependent on contact time to allow RhB molecules to diffuse into the expanded mesopores (4.26 nm) of the modified Laponite. However, at high concentrations, the strong concentration gradient drives rapid initial uptake, causing the surface active sites to saturate quickly. Once saturation is reached, extending the contact time provides negligible benefit, as indicated by the plateau in the upper-right region of the plot.

Figure 2c displays the combined effect of contact time and adsorbent dosage. This plot is particularly useful for process optimization. A broad “high-efficiency zone” (dark green) is observed when the adsorbent dosage exceeds 35 mg and contact time exceeds 120 min. The shape of the contours suggests a synergistic effect; increasing dosage provides the necessary capacity, while sufficient contact time ensures that the intraparticle diffusion resistance is overcome. This interaction defines the operational “design space”, suggesting that a trade-off is possible: the process can achieve similar high efficiencies with a slightly lower adsorbent dosage if the contact time is extended, offering flexibility for economic optimization.

Finally, Figure 2d confirms the validity of the CCD model. The parity plot shows a tight cluster of data points around the diagonal (y = x) line, with no significant outliers. This indicates that the quadratic model successfully captures the complex, nonlinear interactions described above. The high determination coefficient (R^2^ = 98.69%) and the random distribution of residuals confirm that the model is robust and can reliably predict the adsorption performance within the experimental boundaries.

Figure S1 shows the effect of initial pH on removal efficiency. The study found that the removal efficiency of Rhodamine B (RhB) is highly dependent on pH. RhB is a zwitterionic dye that changes its charge and structure with varying pH. Even though both the RhB molecules and the alumina-coated adsorbent surface are positively charged under low pH conditions, leading to an expected electrostatic repulsion, the adsorption efficiency remains high. This suggests that the adsorption mechanism is not solely based on electrostatic forces. Instead, the hydrated alumina surface forms hydrogen bonds with the amine groups of the RhB molecule, overcoming the electrostatic repulsion. As the pH increases, RhB transitions to a neutral or lactonic structure, and the alumina surface becomes negatively charged. This leads to a significant decrease in attractive forces between the dye and the adsorbent, causing a sharp drop in adsorption efficiency.

Considering all these findings, the adsorption mechanism in our study cannot be explained solely by electrostatic attraction; hydrogen bonds, hydrophobic interactions, surface hydration, and surface microstructure also contribute significantly to the process. Contrary to expectations, hydration, particularly on alumina-decorated surfaces, appears to increase adsorption efficiency at low pH. This suggests that surface modifications can have complex, and sometimes even negative, effects on adsorption capacity depending on pH.

### 3.3 Adsorption Isotherms

The experimental adsorption results can be directly correlated with the structural features identified in the characterization analysis. Although the BET surface area decreased upon modification (from 319.0 to 237.1 m^2^/g) due to the blockage of micropores, the adsorption performance remained high (q_max_ ∼ 169.7 mg/g). This apparent contradiction can be explained by the possible evolution of the pore architecture. The Rhodamine B molecule has large molecular dimensions; therefore, its diffusion is often sterically hindered in microporous materials. The Al_2_O_3_ modification expanded the average pore width from 3.07 to 4.26 nm, effectively creating a mesoporous network that facilitates the intraparticle diffusion of the bulky RhB dye molecules into the internal active sites.

Furthermore, the FTIR analysis revealed the emergence of surface hydroxyl groups and Al–O vibrations. These functional groups serve as critical active sites for chemical anchoring. Unlike the purely electrostatic interaction typical of raw Laponite, the Al_2_O_3_-decorated surface enables hydrogen bonding between the surface hydroxyls and the amine/carboxyl groups of the dye, as suggested by the Elovich kinetic model fit (R^2^ = 0.994). Thus, the enhanced performance is a result of the synergistic effect between the optimized mesopore size (physical accessibility) and the introduction of reactive surface functionality.

Various isotherm models have been developed to understand and model adsorption processes. These models allow for the quantitative description of the adsorption process occurring on a solid surface. Isotherm models generally describe the behavior of a system based on many parameters, such as surface properties, the adsorption mechanism, and adsorbent–adsorbent interactions. The most used isotherm models include the Langmuir, Freundlich, Toth, Redlich–Peterson, Sips, and Jovanovich isotherms.^13,14^ Each of these models is built on different assumptions of surface homogeneity, adsorption energy distribution, and single- or multilayer adsorption. In this study, the experimentally obtained adsorption data were compared with these isotherm models, and the agreement between model predictions and experimental results was evaluated using multiple error criteria. The criteria used included R^2^ (coefficient of determination), RMSE (root-mean-square error), MAE (mean absolute error), Hybrid MADQuart (%), Chi-square (χ^2^), and SNE (normalized sum of error).^15,16^ The obtained error values are listed in Table S3.

Additionally, isotherm models were used to analyze the adsorption of the Rhodamine B dye. The comparison of experimental adsorption data with theoretical isotherm models was shown in Figure S2. The Toth and Sips isotherm models provided the best fit for the experimental data, as they are specifically designed to account for heterogeneous adsorbent surfaces and provide accurate predictions across a wide range of concentrations. The low error metrics (RMSE, MAE, χ^2^) and the heterogeneity parameters (t < 1 for Toth) confirmed this finding. While the Langmuir and Redlich–Peterson models also showed a good fit (R^2^ > 0.99), their higher error metrics indicated that they were less accurate than the Toth and Sips models. The Freundlich and Jovanovich models, however, were inadequate in representing the adsorption behavior of the system, showing the worst performance based on their high error metrics and low fit coefficients. Consequently, the models that provided the best fit to the experimental data were Toth, Sips, and Redlich–Peterson, respectively. A common feature of these three models is that they include parameters that account for surface heterogeneity and have effective predictive power over a wide concentration range. While the Langmuir model yielded relatively successful results, it was limited by its ignorance of heterogeneity. The Freundlich and Jovanovich models, on the other hand, were found to be inadequate in representing the true adsorption behavior of the system. These results highlight the importance of considering not only the R^2^ value but also multiple error criteria in model selection.

### 3.4 Adsorption Kinetics

The comparison of experimental adsorption data with theoretical kinetic models can be seen in Figure 3. The PFO kinetic model generally used to describe physical adsorption processes, yielded an adsorption capacity of q_e_ = 104.439 mg/g and a rate constant of k_1_ = 0.098 l/min. This model equation is expressed below. However, its poor agreement with experimental data suggests that the adsorption mechanism cannot be explained solely by physical interactions.

**Figure 3:**
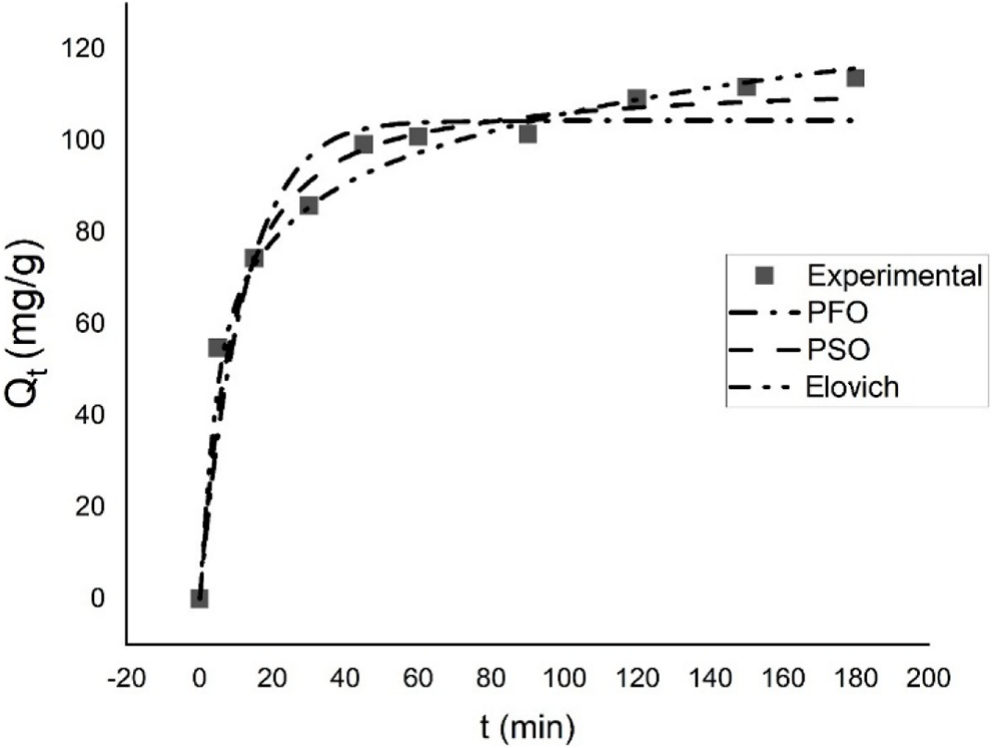
Comparison of experimental adsorption data with theoretical kinetic models.


$$q_{t}=q_{e}\left(1-e^{k_{1}t}\right)$$

The PSO model is generally used to describe chemical adsorption. In this model, the equilibrium capacity was calculated as q_e_ = 113.517 mg/g, and the rate constant was k_2_ = 0.00129 g/mg·min. This model uses a second-order rate expression and specifically considers the filling process of the vacant active sites on the surface. Although the PSO model has reasonable error values, it appears not to adequately reflect the heterogeneous structure of the surface. The PSO model is given by the following equation:

$$q_{t}=\frac{k_{2}q_{e}^{2}t}{1+k_{2}q_{e}t}$$

The Elovich model finds application in situations where the adsorption process occurs on heterogeneous surfaces and active sites with different energies. The model can be described by the following equation:

$$q_{t}=\frac{1}{\beta }\ln\left(\alpha \beta t+1\right)$$

In this equation, α is the initial adsorption rate (mg/g) and β is the resistance coefficient, which depends on the surface’s activation energy. The obtained parameters are α = 100.52 mg/g·min and β = 0.060, indicating that the system initially adsorbed very rapidly and that the surface had reactive sites with low energy barriers. The strong fit obtained by the Elovich model, as indicated by the high R^2^ and low error values, suggests that surface heterogeneity and, possibly, chemical interactions are decisive in the adsorption mechanism.

### 3.5 Adsorption Thermodynamics

The evaluation of thermodynamic parameters is imperative for a comprehensive understanding of adsorption processes. The values of ΔG, ΔH, and ΔS are instrumental in elucidating not only the adsorption tendency but also its underlying mechanism. In this context, van’t Hoff analysis, performed using equilibrium constants derived from the Langmuir isotherm, provides detailed information about the adsorption process (see Figure S3 and Table S5).

In the present study, experiments were conducted at 25, 35, and 45 °C, and van’t Hoff analysis results yielded high linearity (R^2^ = 0.9959). The calculated positive enthalpy change (ΔH = +11.16 kJ/mol) indicates that the adsorption process is endothermic. From a thermodynamic perspective, this finding indicates that the adsorption capacity should increase with increasing temperature. However, experimental findings demonstrated a marginal decline in adsorption capacity with rising temperature. This phenomenon can be elucidated by the observation that, at elevated temperatures, the propensity for desorption from the surface increases in proportion to the increase in the kinetic energy of the molecules. Consequently, low-molecular-weight water molecules within the solution are displaced from the surface and enter the solution phase. This increase in disorder is consistent with a positive entropy change (ΔS = +24.1 J/mol K), thereby creating a predicted outcome.

Within the temperature range that was examined (25–45 °C), the free energy change (ΔG) values were found to be positive, ranging from 3.5 to 4.0 kJ/mol. This outcome indicates that adsorption at low temperatures exhibits limited thermodynamic spontaneity; however, the process possesses the potential to become more favorable with increasing temperature. Consequently, the process is influenced not solely by thermodynamic parameters but also by the impact of surface saturation, desorption rate, and competitive interactions within the solution.

The adsorption behavior observed in this study highlights the complexity of interactions occurring between Rhodamine B molecules and the laponite-based adsorbent. Kinetic analysis revealed that adsorption followed a pattern best represented by the Elovich model, thereby emphasizing the importance of heterogeneous surface sites and varied activation energies. This finding indicates that the adsorbent offers a variety of active sites, resulting in an initial rapid uptake, followed by a subsequent approach toward equilibrium. From a thermodynamic perspective, the process was characterized as endothermic, with relatively low enthalpy values, consistent with physical adsorption. This finding suggests that, while adsorption is energetically favored at higher temperatures, the driving force is relatively weak and susceptible to competition with desorption phenomena as molecular mobility increases. The positive entropy values provide further evidence in support of the hypothesis that adsorption involves structural reorganization at the solid–solution interface, likely through the release of solvent molecules and an increase in system disorder. Taken together, these findings suggest a dual mechanism: specific interactions on high-energy surface sites contribute to the initial rapid uptake, while weaker physical forces dominate the overall adsorption process. This mechanism serves to reconcile the strong kinetic fit with the Elovich model and the thermodynamic indications of physisorption. This interpretation is consistent with the findings of previous studies on dye adsorption onto clay-based materials, where surface heterogeneity and a combination of physical and chemical interactions frequently govern adsorption behavior.

### 3.6 Reusability and Performance Comparison

#### 3.6.1 Desorption and Regeneration Potential

The practical utility of an adsorbent depends on its regeneration capacity and the reversibility of the adsorption process. To evaluate these parameters, we subjected the RhB-loaded Al_2_O_3_-decorated Laponite to various desorption media, including 0.1 M HCl, 0.1 M NaOH, 0.1 M NaCl, and deionized water. As illustrated in Figure 4a, the desorption profiles across all media exhibited a biphasic character: an initial rapid release during the first 10–30 min followed by a gradual approach to a quasi-equilibrium state. This kinetic behavior suggests the presence of two distinct dye fractions: a weakly associated fraction that is easily liberated and a more strongly bound fraction that remains stabilized within the heterogeneous surface framework. Key findings from the desorption studies include: Alkaline Superiority: The highest desorption efficiency occurred in 0.1 M NaOH. In this medium, the deprotonation of surface functional groups induces electrostatic repulsion against the cationic RhB molecules, effectively weakening the surface-dye interactions. Acidic Stabilization: Conversely, desorption was significantly restricted in 0.1 M HCl, indicating that the adsorbed dye layer is highly stabilized under low pH conditions. Ionic Strength Effects: The presence of 0.1 M NaCl slightly enhanced dye release compared to deionized water, likely due to ionic competition and the partial shielding of electrostatic attractions. Mechanistic Correlation: These results corroborate the Toth and Sips isotherm models, which identified a wide distribution of interaction strengths across a heterogeneous surface. While thermodynamic indicators suggested physical adsorption, the limited desorption in neutral and acidic environments points to kinetic barriers, such as molecular reorientation and diffusion into less accessible mesopores (4.26 nm).

**Figure 4:**
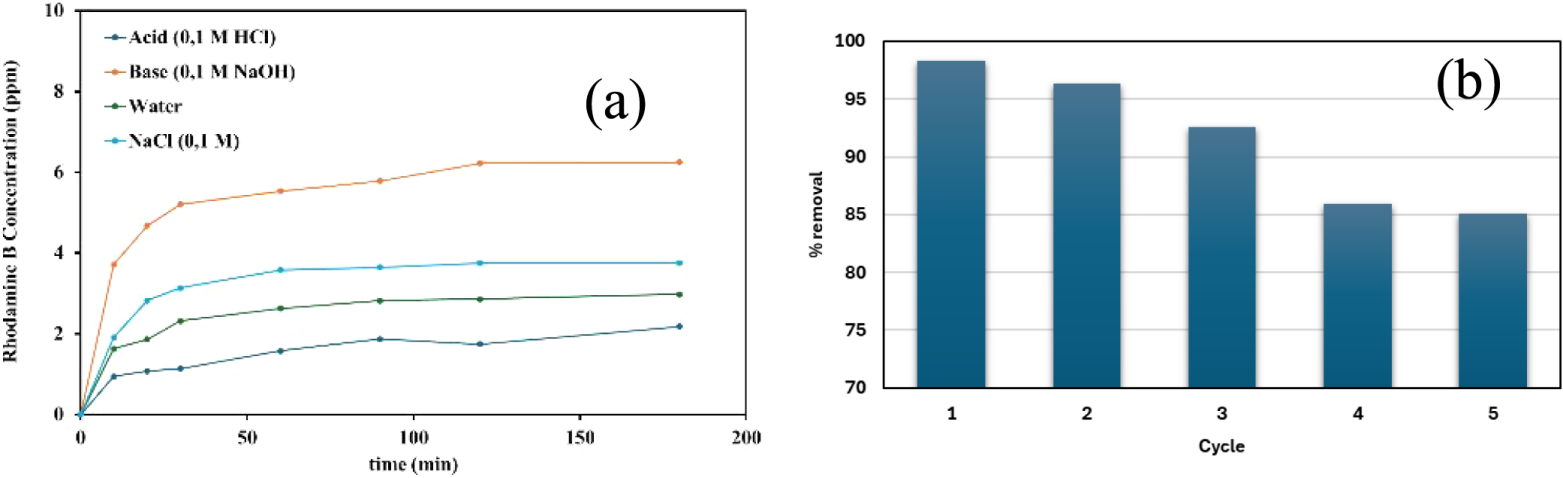
Experimental data for RhB concentration during the desorption experiments (a), reusability chart of the adsorbent (b).

Economic feasibility hinges on adsorbent reusability. Consequently, we subjected the Al_2_O_3_-decorated Laponite to five consecutive adsorption–desorption cycles to assess its industrial potential. Heat treatment was preferred for the recovery of adsorbents, in this study. After the adsorption process, the adsorbents were separated from the solution by centrifugation, dried and heat treated at 400 °C for 3 h. The adsorption process was repeated for the recovered adsorbents, keeping the ratio between the adsorbent and the used solution volume constant. When the system reached equilibrium after 3 h, the amount of RhB was measured with a UV–Vis spectrometer and percentage removal values were determined. The removal efficiency across five consecutive cycles is shown in Figure 4b. The results indicate that the adsorbent retains significant activity, maintaining approximately 85% of its initial removal efficiency after the fifth cycle. This stability suggests that the Al_2_O_3_-decorated Laponite can be successfully regenerated, likely due to the reversibility of the physical interactions (hydrogen bonding) governing the adsorption process.

#### 3.6.2 Comparison with Other Adsorbents

To further assess the performance of the synthesized material, the maximum monolayer adsorption capacity (q_max_) obtained from the Toth model (169.7 mg/g) was compared with other clay-based adsorbents reported in the literature for Rhodamine B removal (Table 2). As seen in the table, the Al_2_O_3_-decorated Laponite exhibits superior or competitive adsorption capacity compared to unmodified clays and other modified composites. This enhanced performance is attributed to the optimized mesoporous structure (4.26 nm average pore width) and the successful introduction of Al–OH active sites, which provide accessible binding sites for the bulky dye molecules.

**Table 2:** Comparison of Maximum Adsorption Capacity (q_max_) of Rhodamine B on Various Adsorbents

Adsorbent Material	q_max_ (mg/g)	Reference
Al_2_O_3_-Decorated Laponite	169.7	This Study
Natural-Bentonite	122.58	^17^
Fe-PILC (Bentonite)	98.62	^18^
Fe_3_O_4_/Al-Clay	62.15	^19^
Moroccon Clay	78.14	^20^
Clay-Cellulose Composite	50.13	^22^
Kaolinite	10	^9^

As presented in Table 2, the Al_2_O_3_-decorated Laponite synthesized in this study exhibits a maximum adsorption capacity (169.7 mg/g) that surpasses most of the commonly used clay minerals such as Bentonite and Kaolinite. This indicates that the decoration of the Laponite surface with alumina not only prevents gelation but also creates a highly accessible mesoporous network favorable for the uptake of bulky dye molecules.

## 4 Conclusions

The present study demonstrated that CTAB/Al_2_O_3_-modified Laponite is a highly effective adsorbent for the removal of Rhodamine B from aqueous solutions. Structural analyses confirmed that modification reduced the BET surface area from 319.0 to 237.1 m^2^/g, while increasing average pore width from 3.07 to 4.26 nm and cumulative pore volume by approximately 32%. The performance of the adsorption process was found to be significantly influenced by the variables employed during its execution. The removal efficiencies attained were found to exceed 92% under conditions of an initial concentration of approximately 50 mg/L, an adsorbent dosage of 30 mg, and a contact time of 100 min. Kinetic studies identified the Elovich model as the most appropriate descriptor (R^2^ = 0.994; RMSE = 2.647; MAE = 1.907), reflecting surface heterogeneity and chemisorption effects. Isotherm analysis demonstrated that the Toth model yielded the most precise representation (q_max_ = 169.7 mg/g; χ^2^ = 2.17; SNE = 0.02), closely followed by the Sips and Redlich–Peterson models. Conversely, Freundlich (R^2^ = 0.942; χ^2^ = 44.1) and Jovanovich (R^2^ = 0.982; χ^2^ = 9.37) models exhibited suboptimal performance. Thermodynamic evaluation confirmed the endothermic nature of adsorption (ΔH = +11.16 kJ/mol) with a positive entropy change (ΔS = +24.1 J/mol·K), indicating increased molecular randomness. Despite the slight positivity of the ΔG values (3.5–4.0 kJ/mol), the adsorption process was found to be favorable, due to surface interactions that extended beyond electrostatics, and which included hydrogen bonding and hydrophobic effects. Collectively, these findings demonstrate that CTAB/Al_2_O_3_-modified Laponite offers a promising and robust adsorbent system for the remediation of dye-contaminated wastewater, combining high adsorption capacity (q_max_ ∼ 170 mg/g) with excellent predictive modeling accuracy.

## Supplementary Material



## Notes

Experimental details; extended characterization data; full ANOVA results; error values for isotherm and kinetic models; thermodynamic analysis tables; supplementary figures (PDF)
