# Supplementary material for: Nonlinear Kinetics and Isotherm Modeling of Rhodamine B Adsorption onto Al2O3 Decorated Laponite
Source: ACS Omega. 2026 Mar 17;11(12):18805–13. doi: 10.1021/acsomega.5c09190 (PMC13044854; doi:10.1021/acsomega.5c09190)
Supplement: Supplementary file 1 [file ao5c09190_si_001.pdf]

Supporting Information for:

NONLINEAR KINETICS AND ISOTHERM MODELING OF RHODAMINE B ADSORPTION ONTO Al_2O_3 DECORATED LAPONITE

[a*]Lütfi ERDEN, [b]İlyas DEVECİ, and [c]Hanife ERDEN

[a] Çanakkale Onsekiz Mart University, Can Vocational School, Department of Electricity and Energy, 17400, Çanakkale, Türkiye

[b] Konya Technical University, Vocational School of Technical Sciences, Chemistry and Chemical Processing Technologies, 42250, Konya, Türkiye

[c] Çanakkale Onsekiz Mart University, Faculty of Engineering, Department of Chemical Engineering,17100, Çanakkale, Türkiye

[*] Email: lutfi.erden@comu.edu.tr

Experimental Details

Instrument models and detailed settings: The structural and morphological properties of the obtained Al2O3 decorated laponite adsorbent were characterized by various analysis techniques. Scanning Electron Microscope (SEM, JEOL JEM-2100) was used to determine the surface morphology of the adsorbent, and Energy Dispersive X-Ray Spectroscopy (EDX) was used to determine the elemental composition. X-Ray Diffraction (XRD, EUROPE 600 Benchtop XRD) analysis was performed to determine the crystal structure changes, and N2 ads/des (MICROMERITICS / TriStar II PLUS) analyses were performed to determine the BET surface area, pore distribution and textural properties.

Detailed experimental procedure: Response Surface Methodology (RSM) was used to investigate the effects of three selected independent variables (concentration, adsorbent dosage and contact time) on the removal efficiency of the model compound. The experimental design was created with a 3-factor Central Composite Design (CCD) approach and planned by Minitab 21 software. A total of 20 experimental conditions were determined, and experimental error was estimated by means of repetitions performed at the central point among these conditions. The independent variables were selected as initial solution concentration (5.0–100.0 mg/L), adsorbent dosage (10–50 mg/L) and contact time (5–180 min). During the experiments, the initial pH value, temperature (25 ± 1 °C) and stirring speed (150 rpm) were kept constant. The removal efficiency (%) was taken as the response variable. According to the determined experimental conditions, an appropriate amount of adsorbent was added to the model pollutant solution prepared and mixed at a preset pH value and constant temperature. At the end of the contact period, the solution was separated by centrifugation and the remaining dye concentration in the solution was measured using a Jenway 7210 model UV-Vis spectrophotometer. The measurements were made at the maximum absorbance wavelength (λmax= 554 nm) of the model pollutant. The removal efficiency was calculated using the following equation:

$$\%R = \frac{C_0 - C_t}{C_0} \times 100$$

In the equation, C_0 represents the initial concentration (mg/L) and C_t is the concentration of the dye remaining in the solution at the end of the contact period determined in the experimental design (mg/L).

Statistical Analysis

Table S1. Results of ANOVA on the Effects of Independent Variables on Adsorption Efficiency

	DF	Adj SS	Adj MS	F-Value	P-Value
Model	9	8954.19	994.91	83.46	0.00000003
Linear	3	4477.73	1492.58	125.20	0.00000003
Concentrations	1	692.27	692.27	58.07	0.00001798

Adsorbent Amount	1	3079.63	3079.63	258.33	0.00000002
Contact time	1	705.82	705.82	59.21	0.00001653
Square	3	2078.30	692.77	58.11	0.00000124
Concentrations*Concentrations	1	125.74	125.74	10.55	0.00875561
Adsorbent Amount*Adsorbent Amount	1	458.80	458.80	38.49	0.00010097
Contact time*Contact time	1	35.85	35.85	3.01	0.11356558
2-Way Interaction	3	2398.17	799.39	67.06	0.00000063
Concentrations*Adsorbent Amount	1	2139.91	2139.91	179.50	0.00000010
Concentrations*Contact time	1	81.68	81.68	6.85	0.02570751
Adsorbent Amount*Contact time	1	176.58	176.58	14.81	0.00321912
Error	10	119.21	11.92		
Lack-of-Fit	5	94.21	18.84	3.77	0.08587658
Pure Error	5	25.00	5.00		
Total	19	9073.41			

According to the ANOVA results, both the linear, quadratic, and most of the interaction terms included in the model are statistically significant ($P < 0.05$) (see Table S1). The effect of the initial dye concentration on % removal is negative and quite significant ($P = 0.000$; $T = -7.62$); this can be explained by the saturation of the active surfaces at high concentrations. The effect of the square of concentration is also significant ($P = 0.009$), indicating nonlinear behavior. Increasing the amount of Alumina Decorated Laponite significantly increases the adsorption efficiency, and this effect is significant at both the linear ($P = 0.000$; $T = 16.07$) and quadratic ($P = 0.000$) levels. Increasing the adsorbent amount provides more active sites, increasing the probability of binding RhB molecules. Contact time also presents a positive and significant effect ($P = 0.000$), but the squared effect of contact time(Saadi et al., 2015) is not statistically significant ($P = 0.114$), suggesting that the effect of duration reaches equilibrium after a certain point and the rate of increase decreases. Interactions between parameters were also found to have an impact on the process. The concentration × adsorbent amount interaction was quite strong ($P = 0.000$), and the combination of these two parameters provided maximum efficiency, particularly in low-concentration-high-adsorbent scenarios. The concentration × contact time interaction was negative and significant ($P = 0.026$), indicating that increasing the time may have limited contribution in high-concentration environments. The adsorbent amount × contact time interaction was also negative and significant ($P = 0.003$), suggesting that the system may reach saturation after a certain point.

Effect of pH on Adsorption Efficiency

Figure S1 shows the effect of initial pH on removal efficiency. Rhodamine B (RhB) is a zwitterionic dye that can undergo structural and charge changes in aqueous solution depending on pH (Rao et al., 2020). This structure allows the molecule to carry both

positive and negatively charged groups simultaneously. Under low pH conditions, Rhodamine B generally exists in its cationic (positively charged) form. As pH increases, the ionization balance between the amine and carboxyl groups within the molecule shifts, leading to a transition to a neutral, even lactonic, structure(Setiawan et al., 2010). This transition is particularly pronounced above pH 9, and the positive charge density in the solution decreases. Consequently, the adsorption capacity decreases significantly.

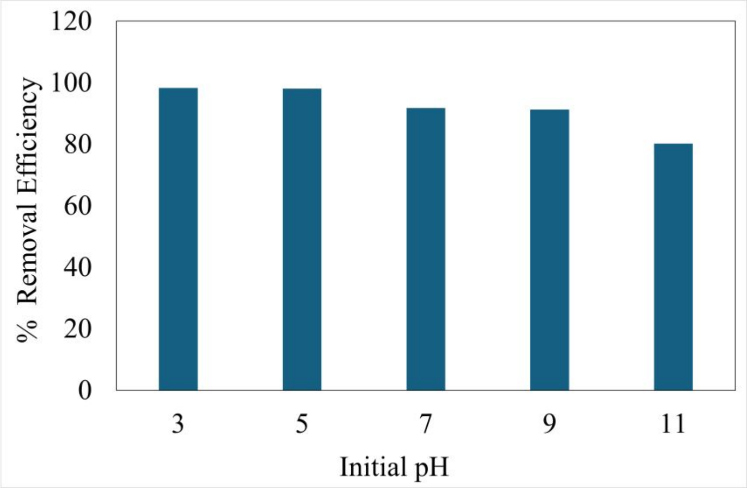

Figure S1. Effect of initial pH on Rhodamine B removal

This behavior is closely related to the pH-dependent charge state of the adsorbent surface. Layered clay minerals such as laponite naturally have negatively charged surfaces, and this charge arises from isomorphic displacements. Therefore, in classical laponite systems, strong electrostatic attractions occur between the positively charged RhB molecules and the negative surfaces, resulting in high adsorption efficiency. However, because the adsorbent surface used in this study was coated with Al_2O_3, the surface chemistry was significantly altered. Alumina (Al_2O_3) is an amphoteric oxide. It interacts with water in low pH environments to form hydroxyl (Al–OH) groups on its surface. These groups can acquire protons and become positively charged. Theoretically, in this case, electrostatic repulsion is expected to occur between the positively charged RhB molecules and the surface, inhibiting adsorption. However, observations have shown that adsorption efficiency remains high under low pH conditions. This demonstrates that the adsorption mechanism is not limited solely to electrostatic forces. At low pH, the hydration of the surface, i.e., the presence of Al–OH groups, increases the ability to form hydrogen bonds with the RhB molecule. Amine groups in the RhB structure can adhere to the surface by hydrogen bonding with hydroxyl groups. Furthermore, the microenvironment created by the hydrated surface facilitates the orientation of amphipathic molecules like RhB to the surface. Such physical interactions suppress electrostatic repulsion, allowing adsorption to remain high. In contrast, as pH increases, Rhodamine B transitions to a neutral or lactonic structure, losing its electrostatic

attraction with the surface. At the same time, the Al_2O_3 surface deprotonates and becomes negatively charged. In this case, the attractive forces between the two molecules weaken. Adsorption efficiency decreases significantly due to RhB becoming hydrophobic and the lower affinity of the alumina surface. Similar findings were observed by Doan and coworkers (Yen Doan et al., 2020).

Adsorption Isotherms

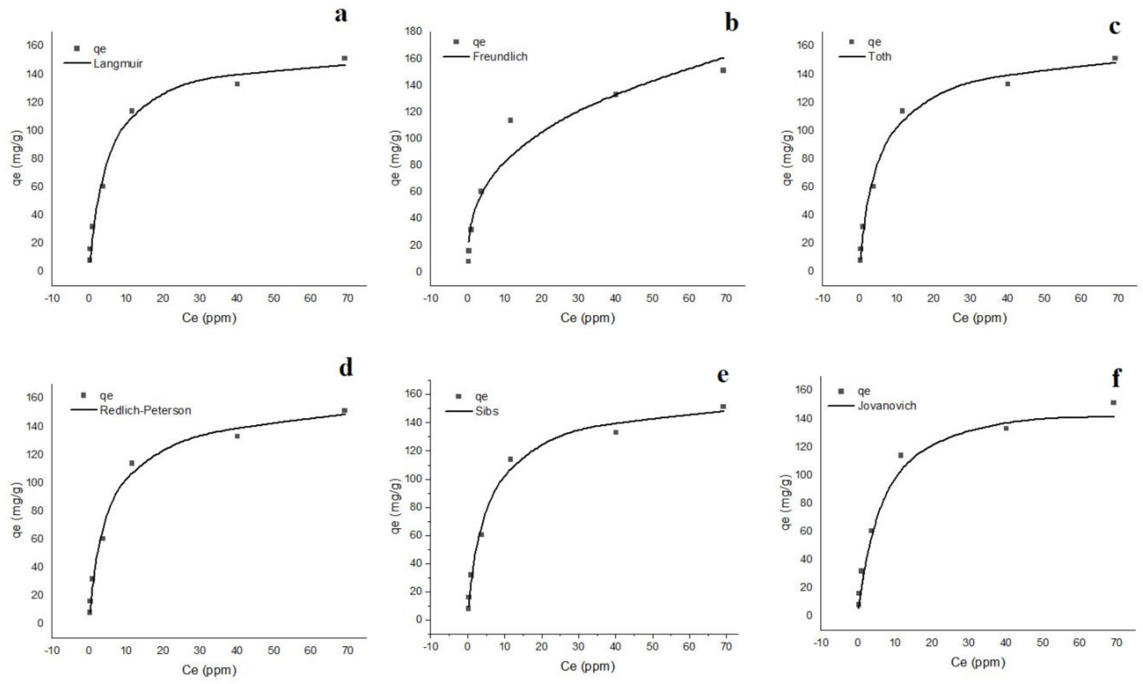

Figure S2. Comparison of experimental adsorption data with theoretical isotherm models: (a) Langmuir, (b) Freundlich, (c) Toth, (d) Redlich–Peterson, (e) Sips, and (f) Jovanovich.

The comparison of experimental adsorption data with theoretical isotherm models are shown in Figure S2. The nonlinear form of isotherm model equations and fitted parameters are also shown in Table S2. The Langmuir isotherm assumes that adsorption occurs only in a single layer on the surface and that all active sites have equivalent energy. Furthermore, it is assumed that a molecule adsorbed at one site does not affect those at neighboring sites. In this respect, the Langmuir model is suitable for homogeneous and ideal surfaces. Compared to experimental data, the Langmuir model exhibited a high coefficient of fit ($R^2 \approx 0.99$) but yielded higher values for error metrics such as RMSE and MAE than the Toth and Sips models. This suggests that the surface may have partially heterogeneous properties. This behavior is illustrated in Figure S2a.

The fitted parameters indicate a relatively high maximum adsorption capacity (qmax=156.962) and moderate affinity constant (K_L=0.200439). These values suggest efficient adsorption but within the assumption of surface homogeneity. The Freundlich isotherm is an empirical model developed for adsorption on heterogeneous surfaces and assumes that adsorption may be multilayered. A key feature of the Freundlich model is that it does not predict maximum adsorption capacity, which can lead to problems with physical significance, especially at high concentrations. In this study, the Freundlich model had the lowest R^2 value but also the poorest fit for other error metrics (RMSE ≈ 13.1, MAE ≈ 10.0, χ^2 ≈ 44.1). This result demonstrates that the system does not conform to the assumptions of the Freundlich model and that this model is inadequate in representing adsorption behavior. The deviation from experimental data is clearly observed in Figure S2b. The low value of 1/n=0.325572 suggests favorable adsorption. However, since the model lacks a saturation limit, it may not describe high concentration regions accurately. In this study, the model showed relatively poor performance, as reflected in error metrics. The Toth isotherm is considered a generalization of the Langmuir model and includes a parameter that considers surface heterogeneity. The Toth model can provide more accurate results than the Langmuir model at low and high concentrations. In this study, the Toth model provided results that were highly consistent with experimental data in both low and high concentration regions, achieving the lowest values for all error metrics (RMSE, MAE, and χ^2,). Furthermore, according to the SNE (Sum of Normalized Errors) criterion, it provided the lowest total error value, providing the best fit with the experimental data. This demonstrates that the Toth model performs superiorly in systems with high adsorbent surface heterogeneity. This excellent fit is presented in Figure S2c. The fitted parameters (q_{max}=169.738, K_T=2.84356, t=0.770018) demonstrate the model's capacity to accurately represent both low and high concentration regions. The value of t < 1 confirms the heterogeneous nature of the adsorbent surface. Among all models, Toth exhibited the best overall performance. The Redlich–Peterson isotherm is a three-parameter hybrid model that incorporates the features of both the Langmuir and Freundlich isotherms. This model is applicable to both homogeneous and heterogeneous systems. In our study, the Redlich–Peterson model yielded very successful results in terms of R^2 (≈0.9925) and ranked just behind the Toth and Sips models in terms of error metrics. The model's high flexibility allowed it to produce predictions consistent with experimental data. The quality of this fit is shown in Figure S2d. The value β=0.9397731 indicates behavior close to the Langmuir model, while the parameter B=0.305419 reflects moderate surface energy variation. This model also provided strong agreement with experimental data. Like the Redlich–Peterson model, the Sips isotherm is a combined form of the Langmuir and Freundlich isotherms. However, it exhibits Langmuir-like behavior, particularly at high concentrations, and Freundlich-like behavior at low concentrations. In this respect, it provides highly consistent results across a wide concentration range. In this study, the Sips model produced error values very close to the Toth model and showed very low deviations in metrics such as RMSE, MAE, and χ^2, demonstrating good fit with the experimental data. It also stood out as the second-best model in terms of SNE values. This demonstrates that the model successfully represents the transition regions during the adsorption process. The agreement between the model and data is evident in Figure S2e. Its parameters (q_{max} = 165.993, K_S = 0.2197, n = 0.858772) suggest Freundlich-like behavior at low concentrations and Langmuir-like saturation at high concentrations. Its flexibility makes it suitable for systems with variable adsorption site energies, and it showed very close performance to the Toth model. The

Jovanovich isotherm describes monolayer adsorption similarly to the Langmuir model, but it also considers the possibility of adsorbed species desorbing from the surface during adsorption. The model is generally developed for physical adsorption processes. In this study, the Jovanovich model exhibited lower fit coefficients and higher error metrics compared to other models, deviating from experimental values, particularly at high concentrations. This demonstrates the model's inadequacy in explaining the adsorption behavior in the system. This limitation is visually reflected in Figure S2f. Although the model includes monolayer adsorption like Langmuir, the parameters (q_{max}=140.864, K_J=0.160764) and model form limit its accuracy, particularly at high concentrations. It showed the weakest fit among the tested models

Nonlinear model equations and fitted parameters: Adsorption isotherm models provide mathematical expressions that describe how the amount of a substance retained on an adsorbent surface (qe) varies depending on the equilibrium concentration (Ce). Figure S2 shows the comparative agreement of the experimental adsorption data with the theoretical results obtained using the Langmuir, Freundlich, Toth, Redlich–Peterson, Sips, and Jovanovich isotherm models. Compared to all models, the Sips, Toth, and Redlich–Peterson models more accurately reflected the experimental data in systems with heterogeneous surfaces. This suggests that the adsorption mechanism is not limited to single-layer homogeneous surface interactions, but that surface heterogeneity and multiple interactions also contribute to the process (Figures S2a–f). Table S2 presents the mathematical forms of the isotherm models used in this study, along with the estimated parameters obtained from nonlinear regression fitting. These models are employed to describe the equilibrium relationship between adsorbate concentration in solution and on the adsorbent surface. Table S3 presents error values of fitting isotherm models with experimental data.

Table S2. Nonlinear form of isotherm model equations and fitted parameters

Isotherm Model	Equation	Estimated Parameters
**Langmuir**	$q_e = \frac{(q_{max} * K_L * C_e)}{(1 + K_L * C_e)}$	q_max = 156.962<br>K_L = 0.200439
**Freundlich**	$q_e = K_F * C_e^{\frac{1}{n}}$	K_F = 40.4018<br>1/n = 0.325572
**Toth**	$q_e = \frac{(q_{max} * C_e)}{(K_T + C_e^{t})^{\frac{1}{t}}}$	q_max = 169.738<br>K_T = 2.84356<br>t = 0.770018
**Redlich–Peterson**	$q_e = \frac{(A * C_e)}{1 + B * C_e^{\beta}}$	A = 37.3405<br>B = 0.305419<br>β = 0.939773
**Sips**	$q_e = \frac{(q_{max} * K_S * C_e^{n})}{1 + K_S * C_e^{n}}$	q_max = 165.993<br>K_S = 0.2197<br>n = 0.858772

**Jovanovich**	$q_e = q_{max} * (1 - \exp(-Kj * C_e))$	q_max = 140.864<br>Kj = 0.160764

Table S3. Error Values of Fitting Isotherm Models with Experimental Data

	**R²**	**RMSE**	**MAE**	**Hybrid (%)**	**Chi-square**	**SNE**
**Toth**	0.9926	4.6772	4.082	8.026	2.1678	0.02
**Sips**	0.9929	4.5878	4.0584	8.5587	2.0786	0.0474
**Redlich--Peterson**	0.9925	4.7112	4.092	7.9012	2.4332	0.0591
**Langmuir**	0.9918	4.9459	4.6003	10.7085	3.4181	0.4972
**Jovanovich**	0.9825	7.2005	6.4274	19.1021	9.3692	1.8412
**Freundlich**	0.9420	13.1112	10.0451	44.2858	44.1198	5

Kinetic error values table:

Pseudo First Order (PFO), Pseudo Second Order (PSO), and Elovich models were applied to evaluate the adsorption kinetics of rhodamine B dye, and the agreement of each model with experimental data was comparatively analyzed. The obtained experimental data and fitted kinetic model graph is seen in Figure 3 in Manuscript. The predictive performance of the models was evaluated using statistical metrics such as coefficient of determination (R^2), mean absolute error (MAE), root mean square error (RMSE), and normalized sum of error (SNE). The results are summarized in Table S4. The highest agreement coefficient ($R^2 = 0.994$), and the lowest RMSE (2.647), MAE (1.907), and SNE (0.208) values were obtained for the Elovich model. This indicates that the Elovich model represents the kinetic behavior of the system more accurately than the other models. The PSO model also provided an acceptable level of agreement ($R^2 = 0.986$) but yielded poorer results than the Elovich model in terms of error metrics. The PFO model, however, showed the lowest fit ($R^2 = 0.945$), inadequately explaining the kinetics of the system.

Table S4. Error Values of Fitting Kinetic Models with Experimental Data

**Model**	**R²**	**RMSE**	**MAE**	**Hybrid**	**Chi-square**	**SNE**
**Elovich**	0.994	2.647	1.907	0.021	0.765	0.208
**PSO**	0.986	3.914	3.211	0.053	1.872	0.390
**PFO**	0.945	7.804	6.488	0.124	5.903	0.786

Thermodynamic Analysis

Table S5. Thermodynamic parameters (ΔG, ΔH, and ΔS) calculated from Langmuir equilibrium constants (KL) at 25, 35, and 45 °C using the van't Hoff equation

Temperature (K)	K_L	1/T	$\ln(K_L)$	ΔG (kJ/mol)	ΔH (kJ/mol)	ΔS j/mol
**298,13**	0,20046	0,00335	-1,60714	3,984	+11,16	+24,13
**308,13**	0,23563	0,00324	-1,44549	3,703		
**318,13**	0,26598	0,00314	-1,32432	3,503		

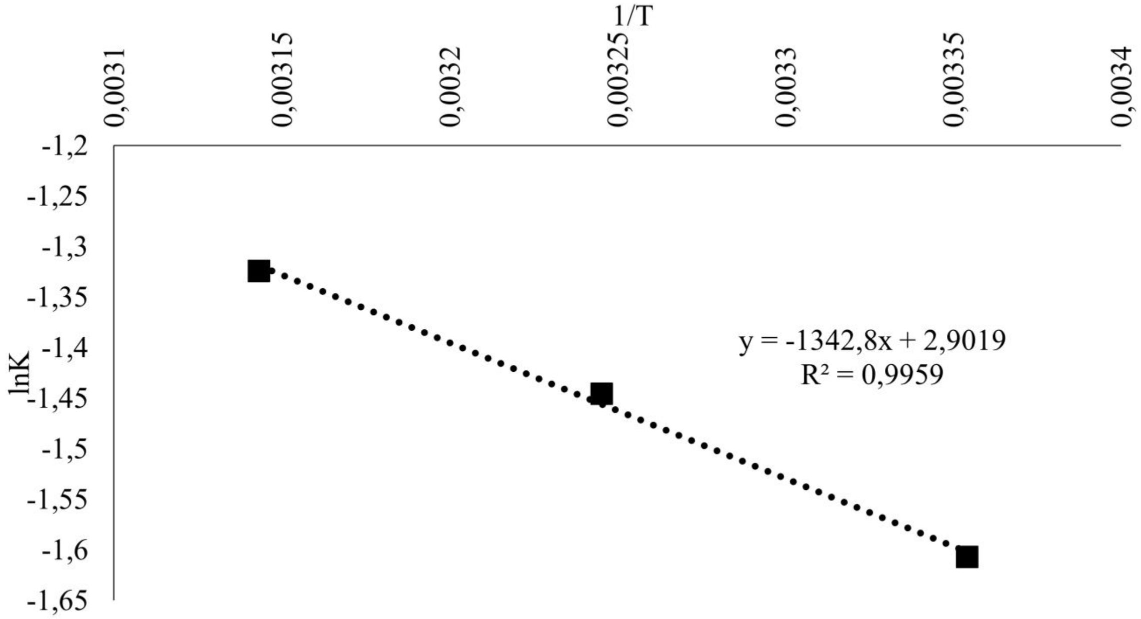

Figure S3. Van't Hoff plot derived from Langmuir equilibrium constants (K_L) at 25, 35, and 45 °C

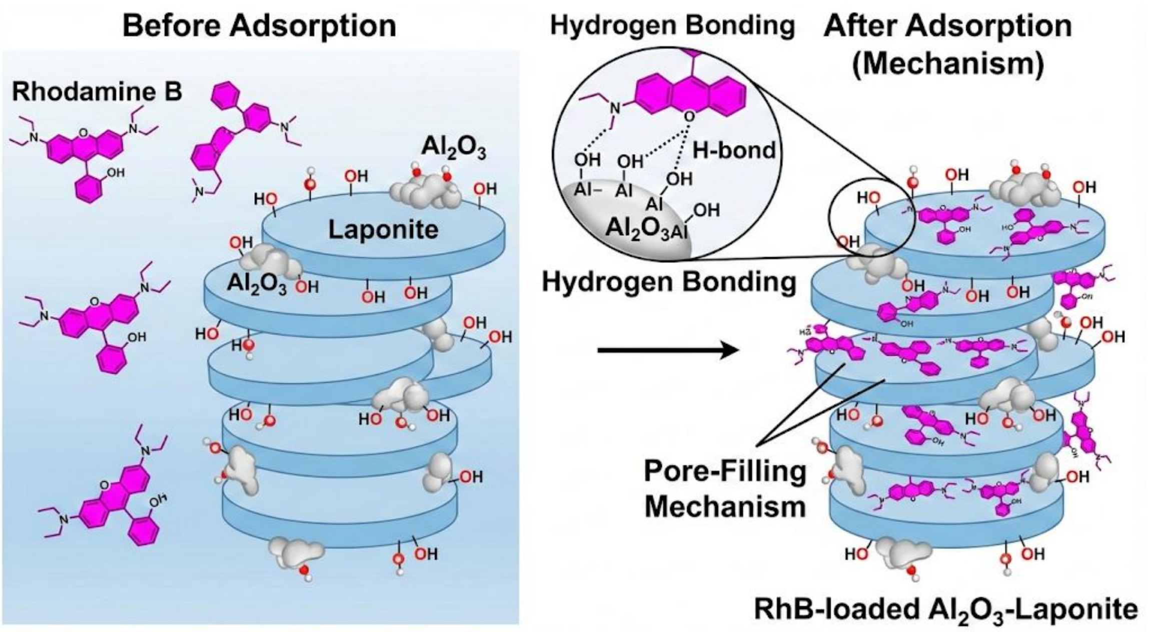

Figure S4. Illustration of the interaction mechanism of RhB and Al_2O_3 surface.

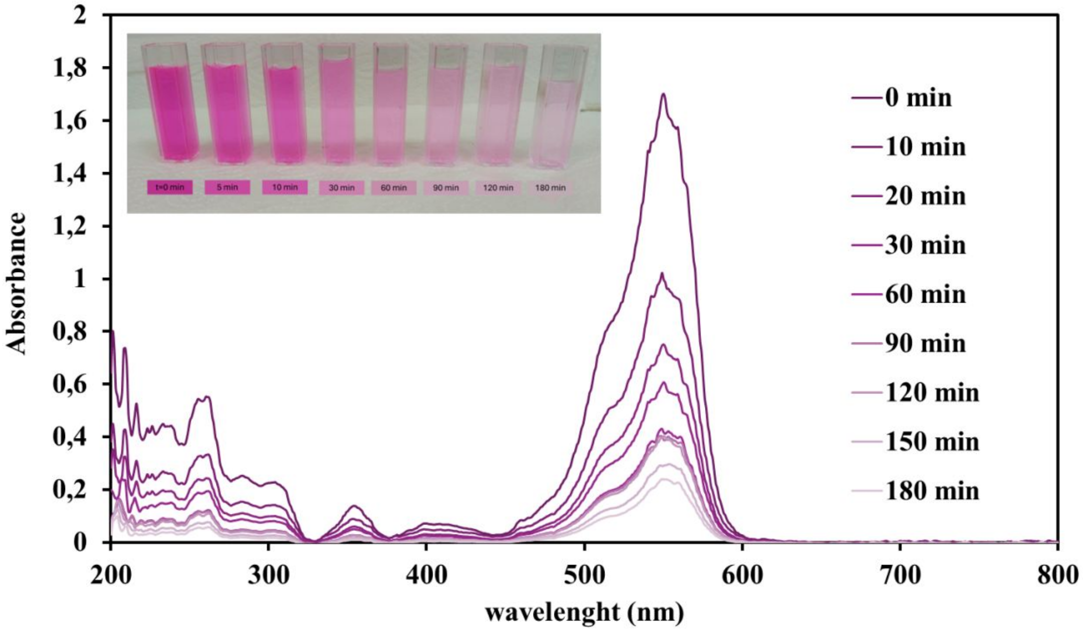

Figure S5. The UV-Vis spectral scan of Rhodamine B before and after adsorption, along with inset photographs showing the visual color change of the solution.
